# Intermittent Character of Certain Diseases of the Nervous System

**Published:** 1846-05

**Authors:** 


*Intermittent character of certain diseases of the Nervous System.*—The tendency to intermittence of the symptoms, in various lesions of the cerebro-spinal system, is a circumstance that ought to be well known to medical practitioners; as they might readily fall into a serious error in their diagnosis, if they were not aware of it. In the acute hydrocephalus of children, the symptoms in the early stage of the disease very usually exhibit more or less of intermittence. I have

---

*** In a case of Scarlatina, related in these reports, Schoenlein advises medical men never to give a confident favorable prognosis in this disease, until the eleventh day is past.**

repeatedly noticed this circumstance in the genuine disease—not to mention that form of fever which has been called *intermittens maligna cerebralis*, and which has been so often mistaken for it. In the after-part of the day, the symptoms, cerebral irritation, usually come on, and continue till about midnight; then they gradually subside, and the child appears to be almost quite well, so that its parents may probably suppose that it was nothing but a casual illness. Again, however, in the afternoon, the child becomes feverish and uneasy; and the same aggravation and subsidence of the symptoms occur as on the preceding day. This state of things will, if unchecked, continue until the pyrexia and head-distress become permanent. When such is the case, mischief has already fairly commenced, and will perhaps utterly defy the best directed efforts of the most experienced physician. In inflammatory affections of the spinal marrow also, this intermittence of symptoms is not unfrequently observed in the early stages of the morbid action.—*Ibid.*

